# A Machine Learning Approach to Perfusion Imaging With Dynamic Susceptibility Contrast MR

**DOI:** 10.3389/fneur.2018.00717

**Published:** 2018-09-04

**Authors:** Richard McKinley, Fan Hung, Roland Wiest, David S. Liebeskind, Fabien Scalzo

**Affiliations:** ^1^Support Center for Advanced Neuroimaging, Inselspital, University of Bern, Bern, Switzerland; ^2^Department of Neurology, University of California, Los Angeles, Los Angeles, CA, United States

**Keywords:** machine learning, stroke, perfusion, reperfusion, penumbra

## Abstract

**Background:** Dynamic susceptibility contrast (DSC) MR perfusion is a frequently-used technique for neurovascular imaging. The progress of a bolus of contrast agent through the tissue of the brain is imaged via a series of T2^*^-weighted MRI scans. Clinically relevant parameters such as blood flow and Tmax can be calculated by deconvolving the contrast-time curves with the bolus shape (arterial input function). In acute stroke, for instance, these parameters may help distinguish between the likely salvageable tissue and irreversibly damaged infarct core. Deconvolution typically relies on singular value decomposition (SVD): however, studies have shown that these algorithms are very sensitive to noise and artifacts present in the image and therefore may introduce distortions that influence the estimated output parameters.

**Methods:** In this work, we present a machine learning approach to the estimation of perfusion parameters in DSC-MRI. Various machine learning models using as input the raw MR source data were trained to reproduce the output of an FDA approved commercial implementation of the SVD deconvolution algorithm. Experiments were conducted to determine the effect of training set size, optimal patch size, and the effect of using different machine-learning models for regression.

**Results:** Model performance increased with training set size, but after 5,000 samples (voxels) this effect was minimal. Models inferring perfusion maps from a 5 by 5 voxel patch outperformed models able to use the information in a single voxel, but larger patches led to worse performance. Random Forest models produced had the lowest root mean squared error, with neural networks performing second best: however, a phantom study revealed that the random forest was highly susceptible to noise levels, while the neural network was more robust.

**Conclusion:** The machine learning-based approach produces estimates of the perfusion parameters invariant to the noise and artifacts that commonly occur as part of MR acquisition. As a result, better robustness to noise is obtained, when evaluated against the FDA approved software on acute stroke patients and simulated phantom data.

## 1. Introduction

Perfusion imaging is a vital tool in clinical neuroimaging, and in particular in the imaging of acute stroke patients. In magnetic resonance imaging, Dynamic Susceptibility Contrast (DSC) MR Perfusion imaging is a modality in which a bolus of contrast agent that reduces the signal intensity of *T*2− and *T*2^*^− weighted images is allowed to perfuse through neural tissue while a series of consecutive MRIs is taken. The signal attenuation resulting from the contrast agent can be used to infer the concentration of contrast agent in the volume over time (1). These concentration-time curves on their own cannot be directly interpreted, but clinically relevant measures such as cerebral blood flow (CBF), cerebral blood volume (CBV), mean transit time (MTT), time-to-peak (TTP), and time-to-maximum (Tmax) can be inferred by deconvolving the arterial input function to obtain the residue function: a curve characterizing blood flow through that volume element. These fluid measurements have been widely used in assessing brain damage, abnormalities, and recovery (2). In acute stroke, treatment selection is performed by comparing the volume of the ischemic core (the tissue undergoing cytotoxic edema) with that of the penumbra: the hypoperfused tissue which is at risk, but which may still be salvaged. Parameters extracted from perfusion imaging are vital for identifying the tissue at risk. Various studies have shown correlations between the perfusion parameters and clinical outcome in terms of Rankin score and Barthel Index (3). Perfusion imaging has also been used to assess collateral circulation and indirectly qualify clinical outcome (4). Transient ischemic attack and internal carotid artery blockage and stenosis are also identifiable with perfusion imaging (5, 6).

The inverse problem of inferring the residue function (and thus the perfusion maps) is ill-conditioned, and standard deconvolution techniques such as singular value decomposition (SVD) are highly susceptible to noise and artifacts in the DSC sequence, causing underestimates for some parameters and overestimates for others (7). Certain techniques have been developed to reduce this problem. A smoother residue function can be achieved through a Gaussian process for deconvolution (GPD) (8), Tikhonov Regularization (9), and a physiological model of microvasculature (10). Attempts to provide better estimates of perfusion parameters have also used Maximum Likelihood Estimation Maximization (ML-EM) (11), and Bayesian estimation (12, 10). These novel algorithms have provided encouraging results to improve the robustness of the deconvolution in the context of DSC. In some instances, however, some of these techniques may not be suitable in the setting of acute stroke due to the increased processing time.

In the clinical setting, perfusion maps are interpreted in two distinct ways: by visual inspection, and by thresholding at standard parameter values. These interpretations are complementary: visual interpretation can provide valuable insight into subtleties of the patient's condition, while thresholding can provide volumetric assessments of the extent of hypoperfusion. For example, a threshold of 6s is used as standard in clinical trials to define the ischemic penumbra (13–15). However, both kinds of interpretation are susceptible to noise. As an alternative to improving the quality of perfusion maps by altering post-processing, several attempts have been made to improve interpretation of standard perfusion maps, moving beyond thresholds to apply machine-learning to standard perfusion maps, identifying tissue-at-risk by learning a mapping from perfusion parameters to tissue risk, as learned from a databank of retrospective cases (16). Other data driven approaches have demonstrated significant improvements in predicting tissue fate based on advanced nonlinear regression (17) and deep learning (18), for example. More recently, Yu et al. presented a model predicting hemorrhagic transformation severity directly from source perfusion imaging [i.e. without first performing deconvolution on the concentration-time curves (19)].

While automated prediction and detection hold enormous promise, interpreting the output of a system derived from machine-learning is often difficult. In particular, clinicians base decisions on the appearance of standard perfusion maps, whose relationship to the outputs of an unfamiliar algorithm may be difficult to discern. To mitigate this, we propose to train a machine-learning models to reconstruct standard perfusion maps from source perfusion, without passing via SVD. The models are trained on a large number of voxels from perfusion imaging in ischemic stroke cases: the variability of these cases allows our models to disregard erroneous measurements and produce better estimates of perfusion parameters, as we demonstrate by synthetically adding noise to both perfusion cases and phantom data.

## 2. DSC MR perfusion imaging

From each voxel, DSC imaging gives rise to a signal intensity time curve. From this curve, a concentration time curve (CTC) of the contrast agent can be computed following the relation: (10):

(1) $$\begin{array}{r} CTC\left(t\right)=\frac{1}{TE}*\log\frac{I\left(t_{0}\right)}{I\left(t\right)}, \end{array}$$

where *TE* is the echo time of the MRI, and *I*(*t*) is the pixel intensity at a pixel as a function of time *t*, and *t*_0_ is the first time of the series. The concentration time curve of a voxel of interest is modeled with the following relation:

(2) $$\begin{array}{r} \kappa CTC\left(t\right)=CBF\int_{0}^{t}AIF\left(\tau \right)R\left(t-\tau \right)d\tau \end{array}$$

Here, κ is a constant dependent on hematocrit levels in the arterioli and the density of brain tissue, CBF is the cerebral blood flow. CTC(t) is modeled as the signal response of the system of neural tissue and vasculature that the contrast agent moves through to reach the voxel of interest. AIF(t), an arterial input function, which is a CTC(t) at the chosen voxel representing the source of incoming contrast agent, is convolved with R(t), which is the impulse response of the system of neural tissue and vasculature. Using the fluid model, it is possible to compute estimates of the desired parameters from the CTC at all voxels. Each pixel's residue function, R(t), can be recovered using deconvolution. The CBF can be computed from Equation (2). CBV is calculated as a ratio between total volume of incoming contrast agent and total volume of contrast agent moving through the voxel of interest.

(3) $$\begin{array}{r} CBV=\frac{\int CTC\left(t\right)dt}{\int AIF\left(t\right)dt} \end{array}$$

Mean Transit Time (MTT) is the average time that blood may spend in the voxel and is computed as:

(4) $$\begin{array}{r} MTT=\frac{CBV}{CBF} \end{array}$$

TTP and Tmax are defined as the times at which the CTC(t) and R(t) respectively reach their maximum and are calculated accordingly.

## 3. Methods

### 3.1. Study design and data acquisition

The study is based on imaging and clinical data from the UCLA stroke registry, a database approved by the internal review board (IRB). All patients included in this study were treated for an acute ischemic stroke at the UCLA Ronald Reagan Hospital in Los Angeles between 2010 and 2016. Inclusion criteria for this study included: (1) Diagnosis of acute ischemic stroke in the middle cerebral artery (MCA) territory or border-zone areas, (2) last known well time within 24 h, (3) MRI of the brain performed before IV-tPA administration or endovascular clot-retrieval therapy. A total of 344 patients (mean age, 61 years; range 13−97; average NIHSS, 14; range 0−38) satisfied the above criteria and underwent MRI using a 1.5 or 3.0 Tesla echo planar MR imaging scanner (Siemens Medical Systems).

### 3.2. Perfusion image processing

Ground truth perfusion maps, used to train and evaluate the predictive models, were computed using Olea Sphere's Perfusion MRI oSVD algorithm (OLEA S.A., La Ciotat, France). To reduce artifacts, motion correction, spatial and temporal smoothing are applied. CTCs are computed from the image intensity curve *I*(*t*) from the DSC MRI's signal intensity at each voxel. An arterial input function (AIF) was also identified from the CTCs of pixels in major arteries, and manually validated by an expert. An oSVD-based deconvolution was used within the Olea software to compute rCBV, rCBF, MTT, TTP, and Tmax. This deconvolution is a cyclic convolution of the AIF and residue function (R) and is represented as a matrix multiplication of the form: *CTC* = *A*×R*CBF, with × as matrix multiplication, and ^*^ as scalar multiplication. Here, CTC and R are represented as column vectors where each component is the function value at a point in time. *A* is the cyclic convolution matrix constructed from the AIF so that matrix multiplication by *A* results in a discrete convolution. SVD is then run to invert *A* and compute R. The perfusion parameters are then computed from R based on its functional form.

### 3.3. Data preparation

To handle the different time resolution of the DSC acquisition across patients, CTCs and AIFs vectors were resized in the temporal domain to a set of 40 values using bicubic interpolation. AIFs used in training were those chosen by Olea Sphere's automatic AIF inference, to ensure that any difference between the output of our models and the OLEA sphere arises from differences in the model, and not on differences in AIF.

The data from each patient was resampled uniformly across the range of the perfusion parameter of interest. The rationale for this was that taking a random subset from the true frequency distribution of the perfusion parameters would bias our function to unevenly represent the full range of the parameters. Lower sections of the brain included many faulty parameter measurements: these slices were excluded from the training data.

### 3.4. Regression of parameters from source perfusion imaging

**Figure 1:**
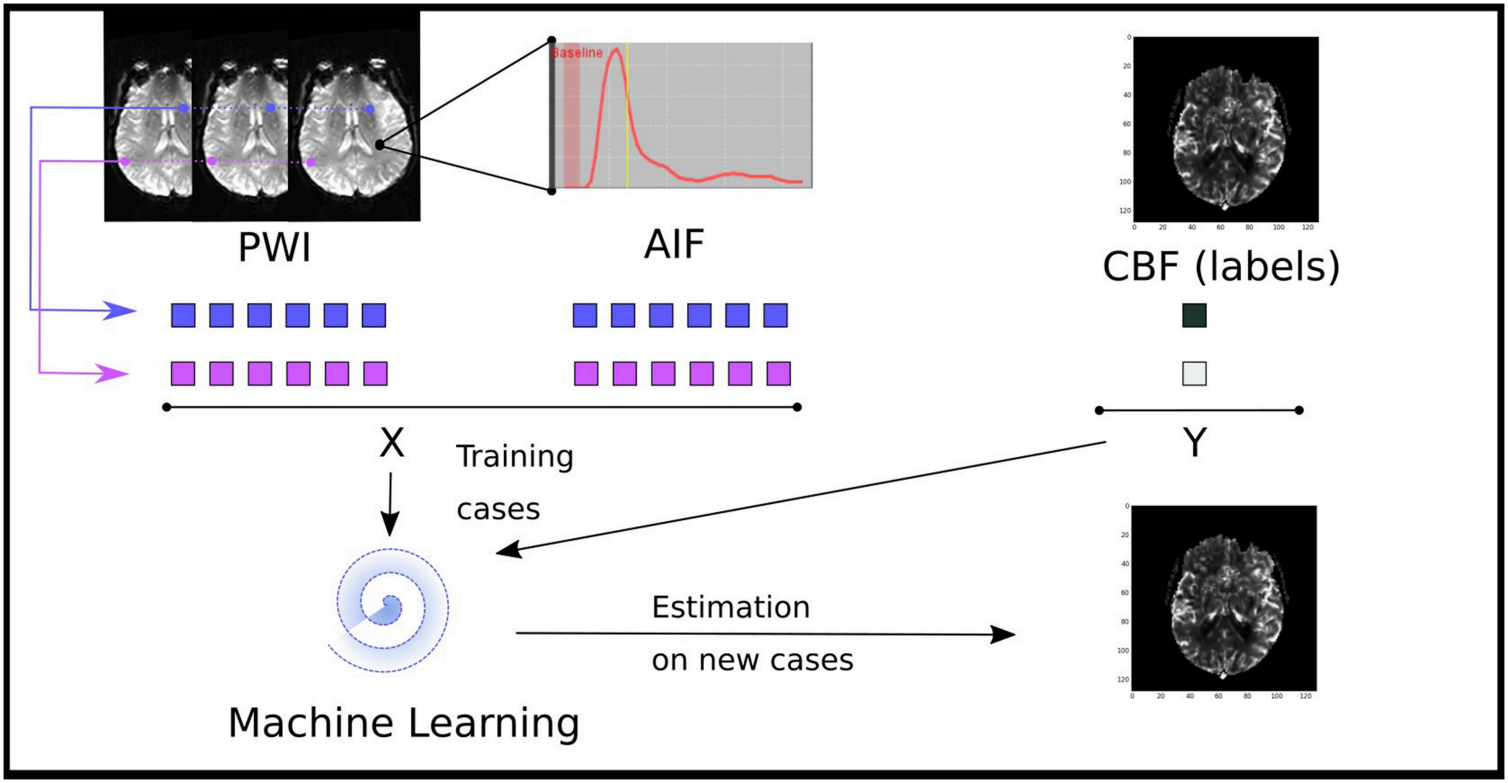
Illustration of the data driven approach to estimate perfusion parameters from a machine learning model trained of the source perfusion MRI data. Supervised learning models are trained to map a pair of a concentration time curve and an AIF to a perfusion map value: these training cases are derived from retrospective stroke cases. Once trained, the performance of the model is assessed on new cases not present in the training data.

We introduce here a regression-based formulation of the reconstruction of perfusion parameters from source MRI images as summarized in Figure 1. The regression model is trained to predict one of the perfusion parameters (i.e., rCBF, rCBV, MTT, Tmax, and TTP) from CTC data at the voxel level. The input to these algorithms takes the form of a one-dimensional vector, containing concentration time curve information combined with the arterial input function. The output of the model is set as the perfusion parameters previously computed used a FDA approved software. All models were trained using Matlab (The MathWorks, Inc., Natick, Massachusetts, United States.)

For each pixel, we define a feature vector, consisting of the concatenation of CTC and AIF data. For a window of size 2*e*+1, the feature vector for a voxel located at the coordinate (*x, y*) is of the form:

(5) () $$x=\left[C_{x-e,y-e},C_{x-(e-1),y-e},\ldots \;,C_{x,y},\ldots ,C_{x+e,y+e},A\right]$$

where *C*_*i, j*_ is a vector of values representing the CTC of the pixel at position (*i, j*):

(6) $$\begin{array}{r} C_{i,j}=C_{i,j,t_{0}},C_{i,j,t_{1}},\ldots ,C_{i,j,t_{n}} \end{array}$$

and *A* is the AIF:

(7) $$\begin{array}{r} A=A_{t_{0}},A_{t_{1}}\ldots A_{t_{n}} \end{array}$$

Note that the explicit spatial/temporal relationships between features is recorded nowhere in the feature vector.

The computation of the value *y*_*i*_ of a perfusion parameter at a given voxel *i* is posed as a regression one, such as *y*_*i*_ = *F*(*x*_*i*_). The function *F* that maps the observed CTC and AIF (*x*_*i*_) to the output parameter is represented by a regression model. When a large number of labeled data points is available, numerous algorithms are available to solve the regression problem. We focus here on standard methods that have been successfully used on a wide variety of applications: support vector machines (SVM), neural network, ridge regression (both linear and kernel), and random forests. In addition, we also include a simple multilinear regression model as baseline method.

#### 3.4.1. Multiple linear regression

Multiple Linear Regression analysis (20) aims at fitting a model such that the sum-of-squares error (SSE) between the observed and the predicted values is minimized. Let β a matrix of *s* parameters,

(8) $$\begin{array}{rcl} Y & = & \beta X+\epsilon \end{array}$$

(9) $$\begin{array}{r} \Leftrightarrow y_{i}=\beta_{1}x_{i}\left(1\right)+\beta_{2}x_{i}\left(2\right)+\ldots +\beta_{s}x_{i}\left(s\right)+\epsilon_{i} \end{array}$$

where *i* = 1…*n* and $\epsilon_{i}=N\left(0,\sigma^{2}\right)$ denotes the noise variables. Multiple Linear Regression analysis finds estimates coefficients $\hat{\beta }$ such that they minimize the sum-of-squares error (SSE) which measures the total error between each prediction and the actual value of the output variable,

(10) $$\begin{array}{r} \hat{\beta }={\text{argmin}}_{\beta }\overset{n}{\underset{i=1}{\sum }}{\left(\beta x_{i}-y_{i}\right)}^{2} \end{array}$$

The optimal $\hat{\beta }$ can be expressed as $\hat{\beta }={\left(XX^{T}\right)}^{-1}X^{T}Y$. We used a QR factorization to obtain estimated regression coefficients $\hat{\beta }$.

#### 3.4.2. Random forests

Decision trees use a hierarchical structure that represents a series of recursive tests performed on the input features to produce an output class. To build the structure of the decision trees, we use the Classification and Regression Trees (CART) algorithm (21). CART is a standard learning algorithm for decision trees based on binary recursive partitioning.

The CART algorithm iterates through three steps to create new nodes in the tree, starting with a single root node:

1. For each input feature *x*_*i*_∈*X*, find the split *s*_*i* = 1…*N*_∈*S* which maximizes the splitting criterion for the current node *t*. $\underset{i,j}{\sum }C\left(i|j\right)p\left(i|t\right)p\left(j|t\right)$ where *C*(*i*|*j*) is the misclassifying cost of a class j sample as a class i sample.
2. Assign the best split *s*_*b*_ ∈ *S* to node *t* which maximizes the splitting criterion.
3. Split node using best node split *s*_*b*_ and repeat until stopping criterion is satisfied.

The procedure expands the tree until the minimum number of samples in a leaf node is reached. After all the terminal nodes are found, the tree acquires its maximum size and can be pruned to produce the final tree. Bootstrap aggregating was then used to generate a forest of 100 decision trees. Predictions were obtained by averaging the output of the trees.

#### 3.4.3. Neural network

A standard feedforward neural network was implemented as baseline technique. It consists of three types of layers: the input layer which is connected to the input features, the hidden layers that are connected in a cascade, and the output layer that is used to produce the output label. Each layer is associated with a transfer function that applies a weight and bias to its input; these parameters are optimized during the training phase of the model. In this study, a total of 7 layers was optimized using a scaled conjugate backpropagation gradient algorithm.

#### 3.4.4. Support vector machines (SVM)

SVM (22) aims at finding the optimal separating hyperplane that minimizes the misclassification rate, while maximizing the sum of distances of the samples from the hyperplane. Formally, this problem amounts at finding the parameter α,

(11) $$\begin{array}{rc} {\text{argmin}}_{\alpha } & \frac{1}{2}\alpha^{T}Q\alpha -e^{T}\\ \text{subject to} & y^{T}\alpha =0\\ 0\leq \alpha_{i}\leq C, & i=1,\ldots ,n \end{array}$$

where *C* is a constant that controls the amount of penalty on the error term during the minimization process, *e* is a vector of all ones, and matrix *Q* defined as:

(12) $$\begin{array}{r} Q_{ij}=y_{i}y_{j}K\left(x_{i},x_{j}\right) \end{array}$$

(13) $$\begin{array}{r} K\left(x_{i},x_{j}\right)=\exp-||x_{i}-x_{j}|{|}^{2}/2\sigma^{2} \end{array}$$

*K* represents a Gaussian kernel that maps samples into another space and σ is the standard deviation of the kernel. After learning, SVM can be used to make predictions on new samples *x* by evaluating the weighted sum of the distances between the sample and each of the training vectors *x*_*t*_ in the kernel space. Class membership probability estimates were obtained using Platt's scaling method (23) which uses logistic regression on the top of the SVM's scores.

#### 3.4.5. Ridge regression

Ridge regression (24) is a standard technique that aims at minimizing the residual sum of square (RSS) to infer the projection vectors *a*:

(14) $$\begin{array}{r} \arg\;\underset{a}{\min}\underset{i=1}{\sum }{\left(y_{i}-a^{T}{\bar{x}}_{i}\right)}^{2}+\alpha ∥a∥ \end{array}$$

where ${\bar{x}}_{i}=x_{i}-\mu$ are the centered data points with respect to mean μ, *y*_*i*_ is the response vector, and α is a regularization factor on the norm of *a*.

The problem can be formulated as

(15) () (K+αI) a=y

where *K* equals to *XX*^*T*^ in linear ridge regression, and a Gaussian kernel projection for kernel ridge regression, *I* is the identity matrix and α > 0 is a regularization parameter. Solving for *a* can be performed using the Cholesky factorization. Because no eigenvector computation is involved, there is a considerable reduction of computational cost while providing nonlinearity.

### 3.5. Experiments

The purpose of the experiments is to examine if the computation of perfusion parameters from DSC imaging can be performed using a regression formulation (section 3.4). Here, we focus on the following parameters: CBF, CBV, MTT, TTP, and Tmax. For each patient, our dataset holds unprocessed, source perfusion MRI scans within 24 h and their corresponding perfusion parameters computed with a FDA-approved commercial software (Olea Sphere from Olea medical). As part of our experiments, we compare the predicted output of the models to the groundtruth. We report the normalized root-mean-square error (NRMSE) and the coefficient of repeatability (CR) which are two recommended techniques to evaluate regression models (section 3.5.1).

In addition to comparing the equivalence between the maps produced by Olea sphere software and the output of the ML framework, we evaluate the robustness of the ML models in the case of known parameter values using a virtual phantom model (section 3.5.4).

#### 3.5.1. Validation and metrics of accuracy

For the purposes of training and validation, a train-test split was used, in which four fifths of the available cases were randomly selected and used to generate training vectors (as described in section 3.4) and the remaining one fifth of cases were used to validate the models, using the accuracy metrics defined in the following paragraph.

The accuracy of each regression model is assessed using the normalized root-mean-square error (NRMSE) and the coeficient of repeatability (CR); two standard metrics of accuracy recommended in such setting (25) in such setting. The NRMSE is defined from the root-mean-square error (RMSE):

(16) $$\begin{array}{r} \mathrm{RMSE}=\sqrt{\frac{\sum_{i=1}^{n}{\left({ŷ}_{i}-y_{i}\right)}^{2}}{n}} \end{array}$$

where *y*_*i*_ is the groundtruth value, ŷ_*i*_ is the predicted output, and *n* is the number of data samples being tested.

(17) $$\begin{array}{r} \mathrm{NRMSE}=\frac{\text{RMSE}}{y_{\max}-y_{\min}} \end{array}$$

where *y*_max_−*y*_min_ represents the range of the output values.

The coefficient of repeatability (CR) originates from the bland-altman (BA) plot which represents the differences between groundtruth (*y*_*i*_) and predictions (ŷ_*i*_):

(18) $$\begin{array}{r} BA\left(x,y\right)=\left(\frac{{ŷ}_{i}-y_{i}}{2},{ŷ}_{i}-y_{i}\right) \end{array}$$

BA captures the error with respect to the value in the output space. It is common to look at the standard deviation within that space; the smaller the standard deviation, the closer the groundtruth and predictions tend to be on average (26). The coefficient of repeatability (CR) precisely captures this notion:

(19) $$\begin{array}{r} CR=1.96\times \sqrt{\frac{\sum {\left({ŷ}_{i}-y_{i}\right)}^{2}}{n}} \end{array}$$

The CR means that the difference between any pairs of prediction, groundtruth is expected to be in the interval [-CR, CR] for 95% of samples.

#### 3.5.2. Training sample

The first experiment evaluates the effect of the number of training samples on the two metrics of accuracy (i.e., NMSRE and CR). A different model is trained for each of the six regression techniques (linear, ridge, kernel ridge, SVM, neural network, random forests) by varying the number of training samples while keeping the test sample fixed. The number of samples using for training the models was generated using a logarithmic distribution *L* ranging from 100 to 16, 000 samples. The NMRSE and CR metrics are reported for each combination of number of samples and regression model, on each PWI modality; CBF, CBV, MTT, Tmax, and TPP.

#### 3.5.3. Patch size

Regression models are evaluated with a patch size varying from 1 × 1, to 17 × 17. Here, we set the number of training samples to 15, 000 samples. Similarly to the evaluation of the number of training samples, we compute the NMRSE and CR error for each combination of regression models, PWI perfusion parameter, and patch size.

#### 3.5.4. CBF phantom model

The CBF phantom model is constructed by selecting 20 patients at random within our cohort. For each patient, the range of CBF values is discretized into 12 bins within the 5th and 95th percentile. The CTC curves of each pixel falling within each bin are used to compute a trimmed mean (by removing the top and lower 10% outliers). At the end of the process, each patient *p* is characterized by 12 average and idealized CTC curves *F*_*p*_ = C_1…12_ together with a manually validated, low-pass filtered AIF curve *A*_*p*_.

To evaluate the robustness of each regression models to noise, the phantom is produced by altering the idealized CTC curves *F*_*p*_ using additive white Gaussian noise (AWGN), ranging from a SNR of 50 to 1 dB. Similarly to our previous experiments, we report the NRMSE as metric of accuracy to correctly estimate the correct CBF. As baseline method, we use a deconvolution method of block-circulant singular value decomposition (cSVD) which is a delay-insensitive method typically used to compute CBF (27).

#### 3.5.5. Computational cost

A crucial aspect of the processing of perfusion-weighted MRI is the time required to compute the maps. While some methods may be more accurate and robust to noise, they might also take a prohibitive time to compute new images. In this experiment, we report the average time to compute a CBF perfusion maps from a 128 × 128 × 40 source PWI. Our goal is to find the method that has the best trade-off between computational cost and accuracy.

## 4. Results

**Figure 2:**
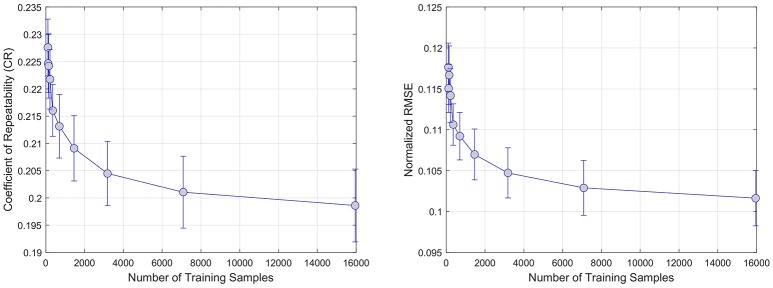
Effect of increasing the number of training samples on error. CR and RMSE are averaged over all modeled modalities (rCBF, rCBV, MTT, Tmax, TTP).

The results of the sample size experiments indicate that both error metrics are decreasing significantly in the first 4, 000 training samples. When looking at the average of all regression models in Figure 2, the reduction goes from 0.225±0.01 to 0.205±0.02 for CR and from 0.12±0.005 to 0.105±0.005 for NRMSE; both are statistically significant reductions (*p* < 0.01).

**Figure 3:**
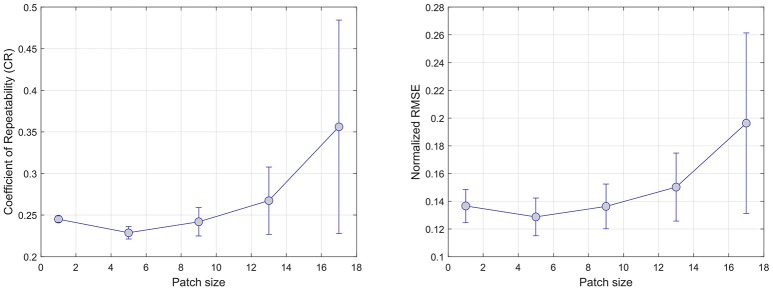
Effect of increasing the patch size on error. CR and RMSE are averaged over all modeled modalities (rCBF, rCBV, MTT, Tmax, and TTP).

Figure 3 summarizes the results of the patch size effect on CR (left) and NRMSE (right) error metrics. In both cases, the average error over all regression models reaches a minimum (CR = 0.23, NRMSE = 0.13) at a size of 5 × 5 pixels. This confirms previous findings (17) where the use regional information using of local patches in the context of regression tend to provide more robust predictions.

Overall results are summarized in Tables 1, 2 for each parameters (TTP, MTT, Tmax, rCBV, rCBF) and each regression model (Linear, Ridge, Kernel ridge, SVM, neural network, random forests). Overall, Random forest was the best method with respect to both error metrics. SVM, Kernel Ridge, and Neural network perform equivalently and can be considered as a good alternative. Linear regression and Ridge regression on the other hand performed more poorly than the rest of the models.

**Table 1:** 15,000 training samples–5 × 5–Normalized Root Mean Square Error ^*^ 100.

**RMSE (STDE)**	**TTP**	**MTT**	**Tmax**	**rCBV**	**rCBF**
Linear	13.28 ± 1.76	12.44 ± 1.54	12.98 ± 1.67	13.06 ± 1.70	12.01 ± 1.43
Ridge	24.51 ± 1.66	15.92 ± 1.39	13.62 ± 1.45	22.30 ± 1.92	20.50 ± 1.42
Kernel Ridge	12.23 ± 1.49	11.47 ± 1.30	12.13 ± 1.46	11.99 ± 1.44	10.89 ± 1.18
Neural Network	11.73 ± 1.35	10.87 ± 1.18	12.05 ± 1.45	11.31 ± 1.28	10.66 ± 1.13
SVM	12.09 ± 1.40	11.46 ± 1.30	12.14 ± 1.41	12.13 ± 1.42	11.26 ± 1.21
Random Forests	10.43 ± 1.08	9.77 ± 0.95	11.29 ± 1.26	10.30 ± 1.06	9.21 ± 0.84

**Table 2:** 15,000 training samples–5 × 5 patch–Coefficient of Repeatability (CR) ^*^ 100.

**CR**	**TTP**	**MTT**	**Tmax**	**rCBV**	**rCBF**
Linear	26.00	24.34	25.35	25.59	23.47
Ridge	25.28	23.13	23.63	27.15	23.37
Kernel Ridge	23.95	22.39	23.67	23.49	21.25
Neural Network	22.79	21.29	23.59	22.14	20.81
SVM	23.23	22.34	23.28	23.33	21.60
Random Forests	20.36	19.07	22.03	20.19	17.99

**Figure 4:**
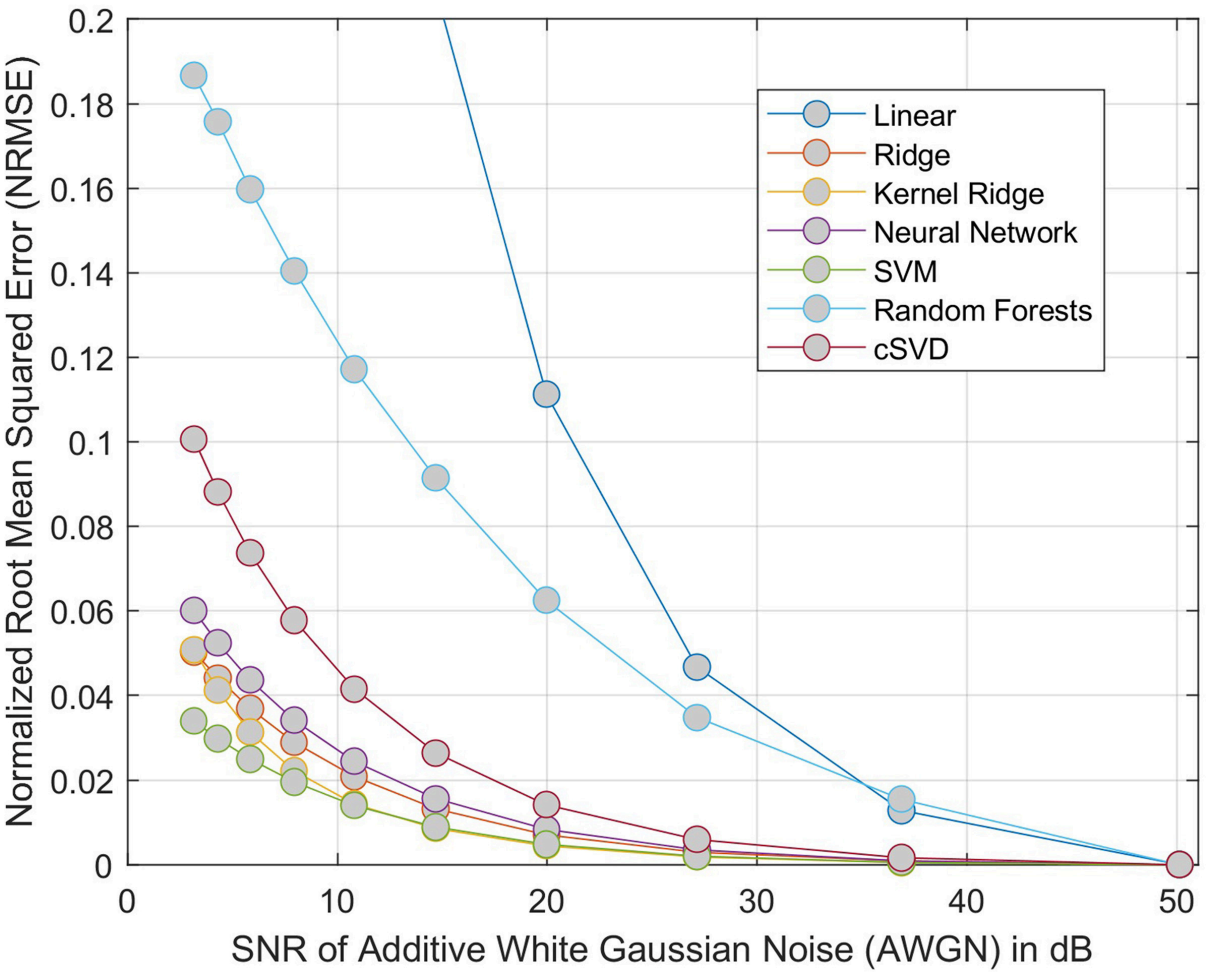
NRMSE of regression-based estimation of rCBF using phantom model, showing the effect of adding noise to the phantom perfusion curves on reconstruction of rCBF. For each model considered, the error is relative to the output of same model trained on data without added noise. The cSVD deconvolution model is used a baseline method.

The results of the phantom experiment are illustrated in Figure 4 where the linear and random forests models do not perform as well as the other methods (including the cSVD deconvolution method). Other regression methods are more stable than cSVD. The differences between these models (SVM, Ridge, Neural Network, Kernel ridge) was not significant. Please note that this result measures the stability of the predictions of each model with respect to noise, but does not reflect the bias associated with the models. The ground truth used here is the output of each model on the idealized curves computed without noise. By adding noise to these idealized curves of known rCBF, we can test if the models produce an estimated output that is similar to what they predict without noise.

In terms of processing speed, linear and ridge regression models perform best with a similar execution time of 0.006 s. Neural network and kernel ridge regression follow with 0.61 and 2.83 s, respectively. Without optimization, SVM and random forests models were more costly with 6.73 7.23 s for predicting a slice of 128 × 128 voxels.

## 5. Discussion

**Figure 5:**
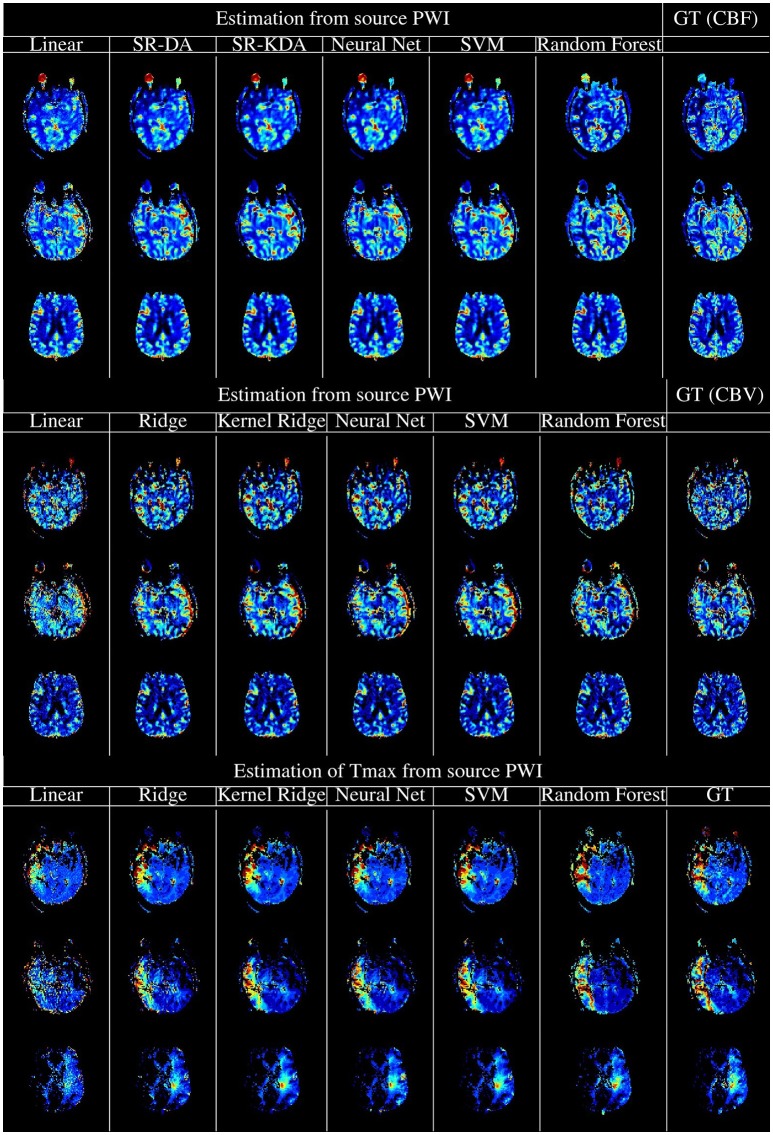
Illustration of the computation of rCBF **(Top)**, rCBV **(Middle)**, and Tmax **(Bottom)** using regression models, vs. the output of oSVD as implemented in OLEA Sphere. From left to right, columns denote the output of linear regression, kernel ridge regression, Feedforward Neural network, support vector machines, random forests, and oSVD as implemented in OLEA (denoted GT for Ground Truth).

The experimental results above demonstrate that machine learning models can produce perfusion maps from source perfusion imaging. The best-performing algorithms in our analysis were neural networks and Random Forests: these techniques produce perfusion maps with similar visual appearance and good numerical correspondence to the output of a standard, FDA-approved perfusion processing algorithm (see Figure 5 for a visual comparison of the machine-learning algorithms and SVD). While Random Forests performed marginally better than neural networks on the original testing data, addition of white Gaussian noise caused this gap to widen significantly, with performance of the random forest model degrading substantially at low SNR, while the neural network model was more robust to noise than SVD. For this reason, overall we judge that the neural network model is the best-performing of the models tested, in terms of ability to reproduce perfusion maps in the presence of noise. Moreover, the neural network model reproduced perfusion maps within less than one second, per slice: given that five perfusion maps must be processed per acquisition, this leads to an unoptimized processing time approximately two minutes per case, which is comfortably within the timescales expected in the clinical setting of acute stroke. Timely, robust processing of perfusion data is vital for assessment and treatment selection in acute stroke: perfusion maps derived from learning algorithms may allow clinical decision making to be made faster, and to more robust to noise in sequence acquisition.

Perfusion-processing typically operates on a voxel-by-voxel level, calculating the perfusion parameters in a voxel from the concentration-time curve of that voxel, together with the arterial input function. Information from neighboring voxels is only incorporated by first applying a spatial smoothing before deconvolution. However, our experiments suggest that robustness of perfusion parameters is improved by incorporating data also from surrounding voxels. This effect is only apparent with small patch sizes. with larger patches leading to an increase in RMSE and CR. This may be a result of the flat data representation used: a model based on, for example, spatial convolutions might be better able to incorporate larger patches without overfitting. The fate of tissue in ischemic stroke is better-predicted from spatial features derived from standard perfusion parameters than from just the voxel-by-voxel parameters (16), but improved spatial perfusion processing incorporating may reduce this effect. Although slice spacing in MR perfusion is rather large (3–6 mm), the use of 3D context (using, for example, three-dimensional convolutional neural networks) may provide further useful information.

The experiments here provide a starting point for the use of other machine learning models on raw CTC data in predicting perfusion parameters as well as us of raw CTC data on predictions in general. One limitation of our approach is that source of the training data, since this is limited to stroke cases from a single center. Further studies would benefit from external validation incorporating multicenter data and covering a number of different pathologies. Perfusion imaging is used, for example, in tumor typing and grading (28, 29). A further limitation of the study is our reliance on an external algorithm for the automatic inference of the arterial input function. This decision allowed us to be certain that, any difference in perfusion maps calculated was due to the machine-learning method, and not a difference in arterial input function. One final limitation of our study is that we do not assess the clinical impact or advantages of the method, concentrating instead on the quality of reconstruction with respect to established methods. A follow-up paper is in preparation which assesses the differences between penumbral volumes, ASPECTS scoring, and eligibility for treatment according to DEFUSE 3, between a machine-learning method and oSVD. Having demonstrated the robustness of machine-learning tools for perfusion analysis to noise, we can, in a further step, analyze the robustness of the generated maps to changes in arterial input function: more ambitiously, we can envisage machine-learning systems which also infer the arterial input function, or even systems which implicitly incorporate arterial blood flow as a latent variable inferred directly from imaging.

As well as reconstructing perfusion maps, there is also potential for these machine learning models to predict other Perfusion MRI related values. In particular, sequence-to-sequence models could be devised to infer the residue function, rather than its related perfusion maps, from the concentration-time curves. Finally, since the goal of perfusion imaging in stroke is to assess the extent of likely tissue damage, we are working currently on models to predict tissue fate directly from source perfusion imaging.

## 6. Conclusion

This paper represents a proof of concept that standard perfusion maps, as used in clinical routine, can be reproduced quickly and with low reconstruction error using simple supervised learning techniques. Nonlinear models such as Random Forests and feedforward neural networks outperformed simpler linear and regularized linear models, and well as kernel-based methods. While the mean squared error of the random forest models were lower than those of the neural network models, the neural network models were more robust to noise. This study paves the way for further advances in the processing of perfusion data by means of machine learning.

## Ethics statement

Study was performed using retrospective data, which was collected from acute ischemic stroke patients admitted at the University of California, Los Angeles Medical Center. The use of this dataset was approved by the local Institutional Review Board (UCLA IRB).

## Author contributions

RM concepted the study, analyzed the data, drafted and edited the article. FH contributed code, drafted and edited the article. RW concepted the study, drafted and edited the article. DL concepted the study, provided data, drafted and edited the article. FS concepted the study, contributed code, analyzed the data, drafted and edited the article.

### Conflict of interest statement

The authors declare that the research was conducted in the absence of any commercial or financial relationships that could be construed as a potential conflict of interest.
